# Reduced subgenomic RNA expression is a molecular indicator of asymptomatic SARS-CoV-2 infection

**DOI:** 10.1038/s43856-021-00034-y

**Published:** 2021-09-22

**Authors:** Chee Hong Wong, Chew Yee Ngan, Rachel L. Goldfeder, Jennifer Idol, Chris Kuhlberg, Rahul Maurya, Kevin Kelly, Gregory Omerza, Nicholas Renzette, Francine De Abreu, Lei Li, Frederick A. Browne, Edison T. Liu, Chia-Lin Wei

**Affiliations:** 1 The Jackson Laboratory for Genomic Medicine, Farmington, CT 06032 USA; 2 Griffin Hospital, Derby, CT 06418 USA

**Keywords:** SARS virus, Infectious diseases, Genomics, Mutation, Transcriptomics

## Abstract

**Background:**

It is estimated that up to 80% of infections caused by severe acute respiratory syndrome coronavirus 2 (SARS-CoV-2) are asymptomatic and asymptomatic patients can still effectively transmit the virus and cause disease. While much of the effort has been placed on decoding single nucleotide variation in SARS-CoV-2 genomes, considerably less is known about their transcript variation and any correlation with clinical severity in human hosts, as defined here by the presence or absence of symptoms.

**Methods:**

To assess viral genomic signatures of disease severity, we conducted a systematic characterization of SARS-CoV-2 transcripts and genetic variants in 81 clinical specimens collected from symptomatic and asymptomatic individuals using multi-scale transcriptomic analyses including amplicon-seq, short-read metatranscriptome and long-read Iso-seq.

**Results:**

Here we show a highly coordinated and consistent pattern of sgRNA expression from individuals with robust SARS-CoV-2 symptomatic infection and their expression is significantly repressed in the asymptomatic infections. We also observe widespread inter- and intra-patient variants in viral RNAs, known as quasispecies frequently found in many RNA viruses. We identify unique sets of deletions preferentially found primarily in symptomatic individuals, with many likely to confer changes in SARS-CoV-2 virulence and host responses. Moreover, these frequently occurring structural variants in SARS-CoV-2 genomes serve as a mechanism to further induce SARS-CoV-2 proteome complexity.

**Conclusions:**

Our results indicate that differential sgRNA expression and structural mutational burden are highly correlated with the clinical severity of SARS-CoV-2 infection. Longitudinally monitoring sgRNA expression and structural diversity could further guide treatment responses, testing strategies, and vaccine development.

## Abstract (web summary)

Wong and Ngan et al. characterize the expression and structural variation of SARS-CoV-2 subgenomic RNAs (sgRNAs) in diagnostic specimens from symptomatic and asymptomatic COVID-19 patients. In a series of genomic and transcriptomic analyses, the authors observe reduced sgRNA expression and a distinct set of structural deletions in asymptomatic infections compared with symptomatic infections.

## Plain Language Summary

Infection with SARS-CoV-2 can lead to symptoms of different severity, with some individuals remaining entirely asymptomatic but still responsible for much of the viral transmission. Here, we sought to identify markers of the severity of symptoms of SARS-CoV-2 infection, as defined by the presence or absence of symptoms. We used a combination of methods to study SARS-CoV-2 genes and their readouts, known as transcripts, in clinical samples from people with symptomatic and asymptomatic infections. We demonstrate that transcripts responsible for making viral proteins are seen at lower levels in asymptomatic infections. We also identify structural changes in the viral genes and transcripts that potentially influence the host’s response to the virus. Our study defines potential markers of symptom severity that may ultimately guide risk mitigation and testing strategies.

## Introduction

COVID-19 emerged in late 2019, was caused by severe acute respiratory syndrome coronavirus 2 (SARS-CoV-2). With its high infectivity and mortality rates, particularly in individuals of older age and those with preexisting health conditions, COVID-19 has rapidly expanded into a global pandemic. Of great importance in the management of the pandemic is the observation that many infected individuals are asymptomatic, ranging from 20–80%^1–3^. Asymptomatic patients, while having faster viral clearance^4–7^, appear to have similar viral loads compared to symptomatic patients^4,5,8–11^ and, therefore, can effectively transmit the disease. Since viral load is not a reliable predictor of disease severity, we examined the genomic biology of SARS-CoV-2 infection in primary patient samples for other correlates of clinical severity, as defined here by the presence or absence of symptoms.

To understand the pathophysiology of COVID-19 infection, a major effort has been made on fully decoding the SARS-CoV-2 genome and its genetic variation, specifically the single nucleotide variants (SNVs)^12,13^ (https://nextstrain.org/sars-cov-2/). SARS-CoV-2 is a positive, single-stranded RNA virus. Upon infecting the host cells, the viruses deploy both replication and transcription to produce full-length (FL) genomic ~30-Kb RNAs (gRNAs) and a distinct set of “spliced” subgenomic transcripts (sgRNAs). These sgRNAs are transcribed through a “discontinuous transcription” mechanism^14^ by which negative-strand RNAs are produced from the 3′ of gRNAs followed by a template switch from a six-nucleotide ACGAAC core transcription regulatory sequence (TRS) that are complementary between 5′ TRS-Leader (TRS-L) and a set of individual TRS-Body (TRS-B) at the 3′-end of the viral genome to join with individual open reading frames (ORFs). For several other nidoviruses, sgRNA transcription may also involve noncanonical TRS motifs^15,16^. These distinct sgRNAs subsequently served as viral mRNAs for translation of multiple structural and accessory proteins including spike surface glycoprotein (S), a small envelope protein (E), matrix protein (M), and nucleocapsid protein (N)^17^. SgRNAs are not packaged into virions and only transcribed in infected cells, therefore, their presence is thought to be an indicator of effective viral replication and in vivo host fitness^18–20^. They are associated with intracellular membranes which protect them from the host and environment-related degradation. Therefore, they can be stably detected in routine diagnostic swabs long after the onset of infection^21^. So far, only a few studies have examined their sgRNAs and they were primarily investigated in in vitro cell culture models^22–24^. Considerably less is known in clinically infected hosts.

Coronaviruses, like many RNA viruses, display high mutations rates caused by the error-prone replication from low fidelity of RNA polymerases^25–27^. Found at variable frequencies, these viral quasispecies accounts for intra-host genetic diversity^28,29^ that contributes to viral adaptation, evolution, and pathogenesis^30–32^. While mostly found as SNVs, studies in in vitro cell-cultured models at high multiplicity of infection (MOI) have demonstrated abundant structural variants^27,33^. These defective RNAs (dRNAs) are found to carry sequence deletions resulted from illegitimate recombination events^25–27^ and associated with disease attenuation^34^. Despite emerging evidence showing the impact of structural variants in the sgRNA coding regions on the severity of infection, transmission rates, and immune responses^35^ (https://www.gov.uk/government/publications/investigation-of-novel-sars-cov-2-variant-variant-of-concern-20201201), SARS-CoV-2 structural variants and sgRNAs, particularly their abundance and complexity in the context host response have been grossly overlooked in the clinical setting. We posited that the molecular characterization of the SARS-CoV-2 associated with asymptomatic infection could help to understand virulence factors contributing to viral pathogenicity and regulation of host responses.

Extended from what has been established through the studies from in vitro virus propagated cell cultures, we systematically characterized the diversity and prevalence of structural deletions and sgRNA expression in human cells collected from swabs for the routine diagnostic purposes from both symptomatic and asymptomatic individuals using a suite of genomic and transcriptomic analyses. From routine swabs collected for diagnostic purpose, we ascertained sgRNA configurations and found that their abundance, both as individual sgRNA species and collectively as a group, is drastically reduced in asymptomatic infection. Moreover, we identified widespread structural deletions in the SARS-CoV-2 genomes, particularly in the regions encoding sgRNAs, consistent to the dRNAs found as a common feature in coronavirus from in vitro studies^34^. Distinct sets of deletions can be consistently and preferentially found in independent SARS-CoV-2 genomes associated with symptomatic and asymptomatic cases, respectively, suggesting their functional significance. To understand the impact of structural variants on viral protein integrity, we analyzed the predicted viral proteomes from FL viral transcript isoforms and supported their validity through proteomic data. Our results reaffirm the highly unstable nature of SARS-CoV-2 subgenomes^33^ and suggest the potential utility of sgRNA expression as an indicator of clinical severity.

## Methods

This study was determined to be exempt from ethical approval by The Jackson Laboratory Institutional Review Board (IRB) because no human sequences was studied in this project. Human sequences were computationally removed, and the remaining reads were mapped to the viral genome. Thus, no human sequences were included in the analytic datasets.

## Sample collection

Samples for the clinical diagnosis purpose were collected by a combination of nasal, oral, nasopharyngeal, and oropharyngeal swabs between April to September 2020. Patient age ranged from 18 to 97 years (median 67 years); 35 were male and 45 were female (Supplementary Data 1). Specimens collected were swabs of nasopharyngeal (*n* = 42), anterior nasal (*n* = 35), and oropharyngeal (*n* = 5). The swabs were preserved in viral transport media were kept at 4–8 °C for less than 72 h between collection and testing. Among them, 51 of the samples were collected from patients presented at the hospital with symptoms consistent with COVID-19. Samples from 30 asymptomatic patients who did not have symptoms consistent with COVID-19 and were collected through contact tracing and workforce screening. All 81 underwent testing through RT-PCR by TaqPath™ COVID-19 Combo Kit (Thermo Fisher) under the FDA Emergency Use Authorization (EUA) with a confirmed positive diagnosis.

### Amplicon sequencing and data processing

Total RNA was extracted from 81 clinical COVID-19 confirmed positive samples using the MagMAX Viral/Pathogen Nucleic Acid Isolation Kit on the KingFisher Flex. The extracted RNAs were used for first-strand cDNA synthesis priming with random hexamer using SuperScript IV as per manufacturers’ instructions. The cDNAs were amplified in two multiplex PCR reactions using the multiplex PCR primers (V3) (Supplementary Data 2) tiled across the viral genome developed by the ARTIC Network (https://www.protocols.io/view/ncov-2019-sequencing-protocol-v3-locost-bh42j8ye) to PCR-amplify the viral genome with primers. The amplicons were pooled and ligated with an Illumina UDI adapter (Illumina). The product were PCR amplified by five cycles and cleaned up using SPRI beads (Beckman Coulter) and subjected to paired-end 300 bp sequencing on Illumina Miseq. Raw paired-end reads were trimmed with trim_galore [https://github.com/FelixKrueger/TrimGalore] (v0.4.3) via cutadapt^36^ (v1.2.1) with the parameters “--stringency 3 -q 30 -e .10 --length 15 --paired”. The trimmed reads were classified with centrifuge-1.0.3-beta^37^ for their potential source. They were aligned to the SAR-Cov-2 reference (MN908947.3) with STAR^38^ (v2.7.3a) with many switches to completely turn off the penalties of noncanonical eukaryotic splicing as documented^23^: “--outFilterType BySJout --outFilterMultimapNmax 20 --alignSJoverhangMin 8 --alignSJDBoverhangMin 1 --outSJfilterOverhangMin 12 12 12 12 --outSJfilterCountUniqueMin 1 1 1 1 --outSJfilterCountTotalMin 1 1 1 1 --outSJfilterDistToOtherSJmin 0 0 0 0 --outFilterMismatchNmax 999 --outFilterMismatchNoverReadLmax 0.04 --scoreGapNoncan -4 --scoreGapATAC -4 --chimOutType Junctions WithinBAM HardClip --chimScoreJunctionNonGTAG 0 --alignSJstitchMismatchNmax -1 -1 -1 -1 --alignIntronMin 20 --alignIntronMax 1000000 --alignMatesGapMax 1000000”. We retained aligned paired-end reads which start with the primer-binding site mutually exclusive from the primers Pool 1 or Pool 2 at the 5′ end of both R/1 and R/2. These retained paired-end reads CIGAR was parsed for jumps and deletions (represented by CIGAR operations N or D of size ≥20 bases).

### SARS-CoV-2 sgRNAs and gRNA expression in the amplicon-seq data

The TRS-L site is located in amplicon 1 of primers Pool 1. Thus, only sgRNAs with TRS-B sites present in the amplicons from primers Pool 1 can be detected. The six detectable sgRNAs are sgRNA_S (Primers 1-and-71), sgRNA_E (Primers 1-and-87), sgRNA_M (Primers 1-and-87), sgRNA_6 (Primers 1-and-89_alt2), sgRNA_7b (Primers 1-and-91), and sgRNA_N (Primers 1-and-93). To classify an aligned paired-end read as originated from sgRNA, it must contain the mentioned primers binding sites from one of the six detectable sgRNAs. Additionally, it must contain at least one split-aligned read that its split read junction marks the leader-to-body junction and that the translated protein product from the concatenated sequence produces the canonical sgRNA. The rest of the amplicon 1 aligned pair-end reads are classified as originated from gRNA.

All sgRNA expression is inter-sample normalized by a scale factor of 1,000,000/total number of mapped read-pairs, giving a comparable measure unit read-pair per million (RPM). The ratio of sgRNA/gRNA is simply computed as the ratio of aligned read-pairs in amplicon 1 as follows: the number of split-aligned read-pairs covering the genomic position 31–75 to the number of read-pairs covering the genomic position 31–410 without split-alignment.

To determine whether any of the identified sgRNAs has resulted from TRS-associated PCR chimera, we examined the amplicon sequencing data produced from the synthetic SARS-CoV-2 RNA Control (TWIST Bioscience #102024). Amplicon sequencing performed on the two biological replicates of human reference RNA spiked in with synthetic SARS-CoV-2 RNA controls^39^ were downloaded from NCBI (SRR13168423 and SRR13168424) and analyzed by the identical processing pipeline. From over 650 K of the sequences aligned to SARS-CoV-2, we did not detect any of the split-mapped reads linking TRS-L and TRS-B.

### Short-read RNA sequencing and data processing

RNA-seq libraries were prepared with KAPA mRNA HyperPrep Kit (Roche) according to the manufacturer’s instruction. First, poly-A^+^ RNA was isolated from 1 ul of total RNA extracted from clinical samples using oligo-dT magnetic beads. Purified RNA was then fragmented at 85 °C for 6 min, targeting fragments range 250–300 bp. Fragmented RNA is reverse transcribed with an incubation of 25 °C for 10 min, 42 °C for 15 min, and an inactivation step at 70 °C for 15 min. This was followed by second-strand synthesis and A-tailing at 16 °C for 30 min and 62 °C for 10 min. A-tailed, double-stranded cDNA fragments were ligated with Illumina-compatible adapters with a unique molecular identifier (UMI) (IDT). Adapter-ligated DNA was purified using Ampure XP beads (Beckman Coulter). This is followed by 17 cycles of PCR amplification. The final library was cleaned up using AMpure XP beads. Quantification of libraries were performed using real-time qPCR (Thermo Fisher). Sequencing was performed on Illumina Novaseq paired-end 149 bases with indexes and nine bases of UMI. Raw paired-end reads were trimmed, potential source classified, and mapped per documented above (Amplicon data processing). Reads deduplication were performed with UMI-tools (v1.0.1)^40^. The aligned paired-end reads CIGAR was parsed for jumps and deletions (represented by CIGAR operations N or D of size ≥20 bases).

### Viral load vs. sgRNA abundance

Samples with ≥100 UMI-deduplicated split-aligned read-pairs are considered (*n* = 45). The sgRNA abundance inter-sample normalized by a scale factor of 1,000,000/total number of UMI-deduplicated mapped read-pairs, giving a comparable measure unit (junction-) RPM. The sample viral load is calculated by transforming the *Ct* value with 2 to the power of (27-*Ct*). The value 27 is chosen to allow calculated values to be comparable to the numbers of junction read per million reads.

### Define genomic RNA and canonical sgRNA reads from Illumina RNA-seq data

We followed^23^ the definition of read classification for sgRNA with a modification. We still required that the split read junction mark the leader-to-body junction and that the translated protein product from the concatenated sequence produces the canonical sgRNA. However, we require that split read 5′ site of deletion is mapped to a genomic position between 59 and 79 (TRS-L: 70–75 nt), instead of 55 and 85^23^. This is established based on the sequence identity between the leader and body regions. For comparable gRNA read count (with respect to sgRNAs read counts), we require that the read must harbor no junction, must overlap the genomic position 1 to 85, and its mate read must be mapped within the first 1000 base of the genome.

The relative abundance of a sample’s sgRNA is, thus, the sgRNA read counts over the sum of the sample’s gRNA and all sgRNAs read count.

### Genomic RNA and canonical sgRNA abundance in Vero cell

DNBseq RNA sequencing data of SARS-CoV-2-infected Vero cell^23^ was downloaded. The data was processed, and expression was computed exactly per our short-read RNA sequencing data.

### Long-read Iso-seq and data processing

Total RNA extracted from nasopharyngeal swabs were prepared according to Iso-seq Express Template Preparation (Pacbio). Full-length cDNA is generated using NEBNext Single Cell/ Low Input cDNA synthesis and Amplification Module in combination with Iso-seq Express Oligo Kit. Amplified cDNA is purified using ProNex beads. For samples with lower than 160 ng in yield, additional PCR cycles is added. cDNA yield of 160–500 ng were then underwent SMRTbell library preparation including a DNA damage repair, end repair, and A-tailing and finally ligated with Overhang Barcoded Adapters. Libraries were then pooled and sequenced on Pacbio Sequel II. The raw sequencing data generated were processed with the SMRT Link (v 8.0.0.80529) Iso-Seq analysis pipeline with the default parameters. Firstly, circular consensus sequences (CCSs) were generated from the raw sequencing reads. Demultiplexed CCSs based on sample barcodes in the adapters, were further classified into full-length, non-chimeric (FLNC) CCSs and non-full-length, non-chimeric CCSs based on the presence of chimera sequence, sequencing primer, and 3′ terminal poly-A sequence. FLNC CCSs (which contains both the 5′-and-3′-adapter sequence along with the poly-A tail) were clustered to generate isoforms. Only the high-quality (accuracy ≥0.99) transcript isoforms (referred here as TUs) were aligned to the SARS-CoV-2 genome reference (MN908947.3) with pbmm2 (v1.1.0). The aligned TU’s CIGAR was parsed for gaps (represented by CIGAR operations N or D of size ≥20 bases). The identified gaps were clustered based on their aligned genomic coordinates. The maximum difference amongst the cluster members’ gap start (and end) coordinates is 10 bases. For TU with multiple transcribed segments, and its first segment 3′ site mapped to the genomic position 59–79, the TU is considered TRS-L mediated. The translation products of the TUs were predicted by translating the sequence with standard genetic code upon the first AUG (Methionine) encountered. The translation product is annotated against a conserved domain database (CDD) including 55,570 position-specific score matrices (PSSMs)^41^.

To determine if there were false-positive deletions resulted from the PacBio sequencing errors, we analyzed PacBio sequencing reads from a 2-Kb synthetic construct as an internal control spiked into the total cDNA templates from the clinical RNA samples. From a total of 3579 CCS reads aligned to this synthetic control reference sequence (GenBank accession MG495226.1) generated in our SARS-CoV-2 Iso-seq PacBio runs, we inspected for the presence of any insertion or deletion using the identical pipeline and filtering criteria (read quality ≥0.99, ≥20 bases) and found none.

### Peptide identification of long-read Iso-seq transcript isoforms translation

Total proteome ThermoFisher RAW files^22^ were downloaded and converted to mzML via ProteoWizard^42^. Peptide spectral matching was performed with Trans Proteomic Pipeline v5.2.0^43^ using the search algorithm Comet v2020.01 rev. 3^44^ with automatic decoy peptides generation against the UniProt Chlorocebus sabaeus database (downloaded March 2021; 19,525 sequences), and a custom SARS-CoV-2 protein database containing both the translation product from NCBI GenBank: MN908947.3 and the PacBio FL transcript isoforms (608 sequences) from this study. The peptide assignments were filtered with PeptideProphet^45^ at FDR <5%, and only assignments to SARS-CoV-2 proteins were summarized.

## Results

### SgRNA expression is drastically repressed in asymptomatic SARS-CoV-2 infection

**Fig. 1:**
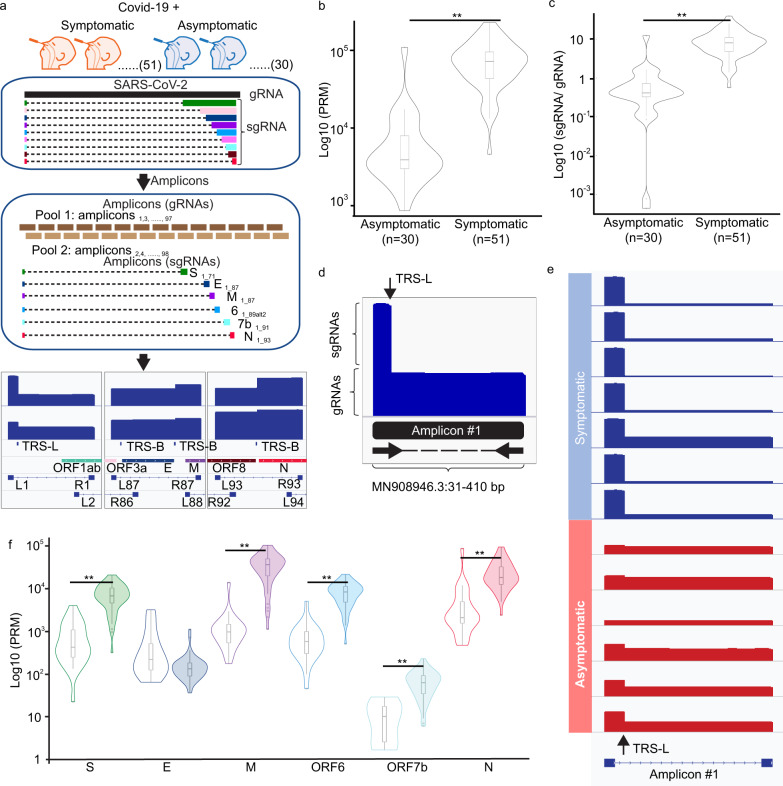
Amplicon-seq analysis characterize sgRNA relative abundance in clinical swabs in COVID-19 positive patients. **a** SARS-CoV-2 RNAs existed in the upper respiratory tracts from both symptomatic and asymptomatic COVID-19 positive patients were analyzed by Amplicon-seq. Amplicons tilted across full-length SARS-CoV-2 genomes were amplified by specific primers in the multiplex PCRs in two separate pools. Subgenomic RNAs (sgRNAs) specific amplicons can be identified by specific amplification with 5′ forward primer closest to the transcription regulatory sequence leader (TRS-L) site and 3′ reverse primers closest to the TRS-Body (TRS-B) sites followed by sequencing and alignment. Distribution of sgRNAs normalized counts (**b**) and sgRNA to genomic RNA (gRNA) ratio (*p* = 4.9  × 10^−12^) (**c**) between symptomatic and asymptomatic cases (*p* = 5.6 × 10^−12^). **d** Sequencing coverage across the 5′ 400 nucleotides of SARS-CoV-2 genome shows the contribution from sgRNAs and gRNA, respectively. **e** Amplicon-seq coverages across 5′ 400 nucleotides from representative symptomatic and asymptomatic cases. **f** Distribution of the normalized counts of individual sgRNA species measured in symptomatic and asymptomatic cases (*P* values of pairwise comparison for S, M, ORF6, ORF7b, and N are 6 × 10^−11^, 9 × 10^−12^, 2 × 10^−11^, 2 × 10^−7^, and 9 × 10^−10^). All statistical tests are two-sided Wilcoxon rank-sum test. Center line, median; boxes, first and third quartiles; whiskers, 1.5× the interquartile range.

SARS-CoV-2 gRNAs and sgRNAs have an overall high sequence identity. To discern sgRNA from the gRNAs, we exploited the features derived from the discontinuous transcription, namely the joining between TRS-L and TRS-B regions whose presence exclusively was found in sgRNAs. We adopted amplicon-based sequencing (amplicon-seq), a method widely used to characterize SARS-CoV-2 genomes^46^, to characterize the presence of sgRNAs and correlate their abundance in the COVID-19 positive samples between symptomatic and asymptomatic patients. Amplicon-seq is highly sensitive, with a limit of detection (LoD) reported as low as one SARS-CoV-2 copy per microliter using the optimized protocols from the Artic network^47,48^. Therefore, it can effectively enrich SARS-CoV-2 cDNAs from samples of a wide range of viral content. In this approach, viral-specific primers were designed across the full-length RNAs and amplicons specific for SARS-CoV-2 sgRNAs can be PCR amplified by 5′ most primer next to the TRS-L sequence as forward primer and reverse primers nearest to the TRS-B sequences in the multiplex PCRs. Based on the locations of primers, we anticipated that amplicons for six out of the nine sgRNA species (sgRNA_S, E, M, 6, 7b, and N) can be found in the amplicon-seq (Methods). Followed by massively parallel sequencing, these subgenomic-specific amplicons can be identified through the junction reads linking TRS-L and TRS-B in the sequencing data and used to determine the relative abundance of sgRNAs (Fig. 1a).

From 51 and 30 SARS-CoV-2 positive symptomatic and asymptomatic patients, respectively (where asymptomatic patients are defined as those who showed none of the key COVID-19 symptoms within 14 days of testing) (Supplementary Data 1 and Fig. S1 displays the age distribution of patients), we extracted total RNA from swabs of different locations of respiratory tracts including anterior nasal, oro- and nasopharyngeal collected for the purpose of diagnostic RT-PCR and performed amplicon-seq to generate deep sequencing data for each sample (>200,000 paired reads, >4000-fold genome coverage) (Supplementary Data 3). From the reads aligned to the reference MN908947.3, amplicon corresponding to six sgRNAs were detected through split-mapped reads connecting the first 75 nucleotides harboring TRS-L sequences to their respective TRS-B sites. The specificity of the sgRNA detection by aligning reads linking TRS-L/B was further verified using amplicon sequencing data produced from the synthetic SARS-CoV-2 RNA control^39^. None of the 650 K of the sequences aligned to SARS-CoV-2 was found with TRS-L and TRS-B connection. To evaluate their relative abundance among different samples, we normalized the amounts of TRS-L associated junction reads against the total numbers of SARS-CoV-2 reads in each sample. Through the normalized junction read counts, we found that the levels of sgRNAs were highly variable, ranging between 0 to 230,155 reads per million (RPM). Between COVID-19 positive individuals with and without symptoms, sgRNA levels were significantly lower in asymptomatic than in the symptomatic samples (median value 3498 vs. 72,231; two-sided Wilcoxon rank-sum test, *p* = 4.9 × 10^−12^) (Fig. 1b). To ensure that the reduction of sgRNA expression was not resulted from potential lower viral load found in the asymptomatic samples, we further compared the expression of sgRNA per viral gRNA (sgRNA/gRNA) in the asymptomatic vs. symptomatic infections. Here, the levels of gRNAs were defined as the number of reads aligned uninterrupted across the first 400 nucleotides because their existence was exclusively found in the viral gRNA molecules. As shown in Fig. 1c, a significantly lower ratio of sgRNA/gRNA (19-fold in median value, two-sided Wilcoxon rank-sum test, *p* = 5.6 × 10^−12^) was observed in asymptomatic hosts, suggesting the lower levels of sgRNAs were independent of virus quantity in these samples. The differences in sgRNA abundance were independent of the sample collection methods as significant lower sgRNA/gRNA ratio were observed in asymptomatic specimens collected by nasopharynx (pairwise Wilcoxon rank-sum test with Bonferroni correction, *p* = 8.9 × 10^−7^) or anterior nasal (*p* = 1.0 × 10^−4^) (Fig. S2). The relative abundance of sgRNAs to gRNAs can also be reflected through the read coverage along with the first 400 nucleotides (Fig. 1d). Here, the distinct differences of sgRNA/gRNA ratio can be observed by the apparent degrees of differential coverage from the first 75 nucleotides (present in both sgRNAs and gRNAs) to the 76–400 nucleotides (only present in gRNAs) visualized through the integrated genomics viewer (IGV) (Fig. 1e), which clearly indicated the existence of the higher amount of sgRNAs in the symptomatic samples.

To evaluate if the reduction of sgRNAs occurred selectively in specific sgRNA species or broadly to all sgRNA transcription, we further compared the levels of each gRNA species detected between symptomatic vs. asymptomatic samples. The expression levels of individual sgRNA species were determined by assigning each TRS-associated junction read to their respective sgRNA origins based on their corresponding TRS-B site usage. Among the six sgRNA-specific amplicons produced in the amplicon-seq, all but one (sgRNA_E) displayed significant reduction (two-sided Wilcoxon rank-sum tests, *p* values 2 × 10^−7^ to 9 × 10^−12^) (Fig. 1f). Among them, sgRNA_M exhibited the highest degrees (6–37-fold) of decline. Collectively, these results indicated that the lack of active viral transcription in the asymptomatic infection and the sgRNA to gRNA ratio in the host cells appears to reflect the degree of disease severity.

### Coordinated expression of sgRNAs in primary human cells of symptomatic infection

**Fig. 2:**
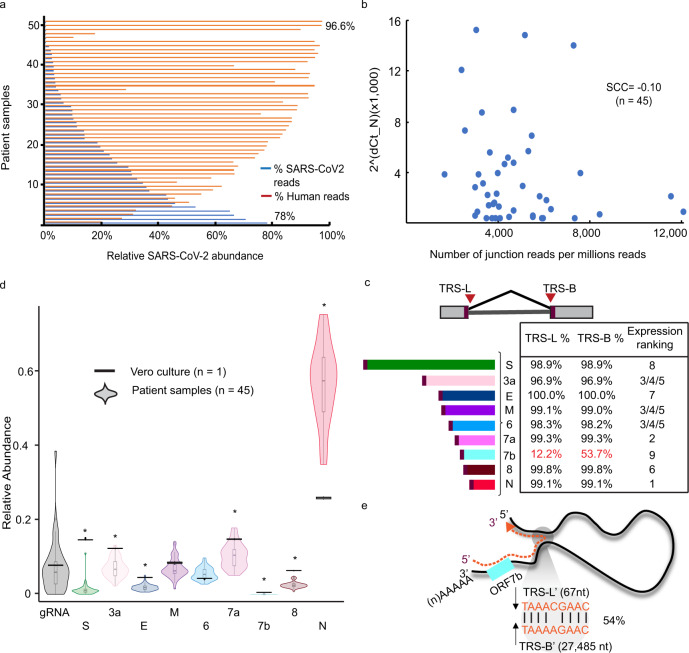
Expression of subgenomic RNAs (sgRNAs) in the clinical specimens from symptomatic patients. **a** The percentages of SARS-CoV-2 (blue) and human (red) reads detected in each of the symptomatic samples (*n* = 51). **b** Correlation analysis between viral load (RT-qPCR *Ct* values) and sgRNA abundance (numbers of junction reads per million). **c** Transcription regulatory sequence (TRS) usage. Percentages of sgRNA-derived junction reads split at their corresponding known TRS-Leader (TRS-L) and TRS-Body (TRS-B) sites for each sgRNA species and the relative abundance ranking. **d** Proportions of reads assigned to genomic RNA (gRNA) and each sgRNA species in symptomatic samples (*n* = 45) and Vero cultured cells (*n* = 1). Center line, median; boxes, first and third quartiles; whiskers, 1.5× the interquartile range; points, outliers. **e** Sequences at the alternative TRS-B sites used by sgRNA_ORF7b transcription.

The differential sgRNA abundance detected in COVID-19 positive samples between symptomatic and asymptomatic patients implicates their potential function in eliciting host responses. To characterize their expression in the infected cells of symptomatic patients, we adopted an unbiased metagenomic RNA-seq approach to survey the types of sgRNAs expressed and quantitatively evaluate their relative abundance in these samples (Supplementary Data 4). In metagenomic RNA-seq analysis, both host and SARS-CoV-2 RNAs expressed were comprehensively revealed by the sequencing of the extracted total RNAs. Using the centrifuge algorithm^37^, we conducted full metagenome profiling and taxonomy classification to assess their relative ratio between humans and SARS-CoV-2. Despite their relatively low *Ct* values (13–19), suggesting high viral content, the ratio of reads aligned to SARS-Cov-2 were highly variable among these samples, ranging from 0.06 to 78% (Fig. 2a). Such high variation of viral to host RNA ratio was possibly influenced by the sampling techniques or the stages of the infection.

We next characterized the types and abundance of sgRNAs expressed in these samples. The TRS-L associated RNA-seq reads were assigned to each of the nine distinct sgRNA species based on their spans across the corresponding TRS-B junction sites closest to the annotated transcript initiation sites. The abundance of SARS-CoV-2 sgRNAs had no correlation with the viral load inferred by the *Ct* values from RT-qPCR testing (Spearman correlation coefficient = −0.10, *p* = 0.50) (Fig. 2b), suggesting that the viral nucleic acid shedding measured by the RT-qPCR diagnostic assays does not reflect the activity of viral replication in these samples. The relative abundance of different sgRNA species exhibited a remarkable consistency both in their expression ranking (Fig. 2c) and the relative proportion of the reads for each sgRNA class (Fig. 2d). Across all samples. SgRNA_N was expressed the highest and sgRNA_ORF7b was the least abundant. It is worth noting that sgRNA_ORF7b was not previously detected in in vitro infected cell cultures^24^. The low expression of sgRNA_ORF7b could be resulted from the imprecision of TRS usage in the discontinuous transcription process. Unlike the other sgRNA species which were mostly transcribed from the annotated TRS sites, 54% of the sgRNA_7b transcripts have adopted an alternative TRS-B′ site MN908947.3:27485 (Fig. 2e). These observations suggested that ORF7b expression is subjected to the high variability and could be dispensable in vivo.

When comparing the relative abundance of sgRNAs to these reported from in vitro Vero cells experiments, seven sgRNAs exhibited significant difference (*p* value < 1e-05) with the most striking difference found in the sgRNA_Spike (S) (Fig. 2d). In primary human samples, sgRNA_S expressed at less than 1% of total sgRNAs but was found at 14% of total expressed sgRNAs in the cultured Vero cells. The discrepancy observed between clinical specimens and laboratory cultured cells could be contributed from multiple sources, including the variation in RNA quality, transport, and storage and the difference could be contributed by the differences in SARS-CoV-2 transmission and entry between the in vitro cell cultures and primary tissues. The expression of sgRNA_ORF10 was not detected, consistent with what has been described in SARS-CoV-2 infected cell cultures^23^.

### Distinct sets of deletions detected in SARS-CoV-2 RNAs from primary human cells between symptomatic and asymptomatic infections

**Fig. 3:**
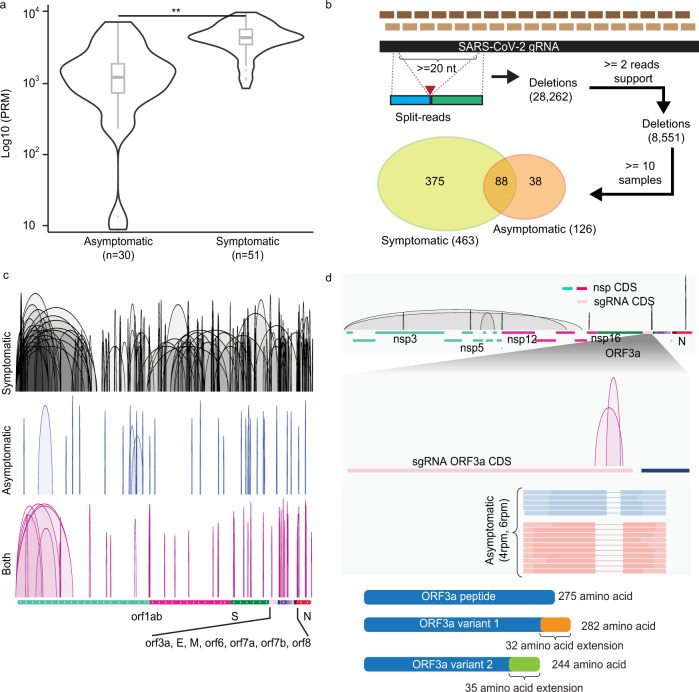
Deletions of SARS-CoV-2 RNAs in symptomatic and asymptomatic COVID-19 positive patients. **a** Distributions of normalized split-aligned reads counts in asymptomatic and symptomatic patients. Two-sided Wilcoxon rank-sum test, *p* = 2.3 × 10^−8^. Center line, median; boxes, first and third quartiles; whiskers, 1.5× the interquartile range. **b** Deletions inferred by amplicon-seq data from asymptomatic and symptomatic patients’ specimens. **c** Visualization of the deletions detected in symptomatic (*n* = 287), asymptomatic (*n* = 34), and both (*n* = 79) samples in IGV genome browser in reference annotated subgenomic RNA (sgRNA) transcribed regions. **d** Top: Deletions (*n* = 10) preferentially found in viral RNAs from the asymptomatic samples. Middle: zoom-in view in sgRNA_ORF3a coding sequence (CDS) region shows the two deletions uniquely found in asymptomatic cases, their normalized counts, and representative read supports. Lower: their predicted translated peptide in reference to the wild-type ORF3a peptide.

It has been reported that novel deletions in sgRNAs may have an impact on the clinical presentation of SARS-CoV-2 infection^35^ and transmission rate (https://www.cdc.gov/coronavirus/2019-ncov/more/scientific-brief-emerging-variant.html; https://virological.org/t/preliminary-genomic-characterisation-of-an-emergent-sars-cov-2-lineage-in-the-uk-defined-by-a-novel-set-of-spike-mutations/563). We, therefore, examined the structural deletions in SARS-CoV-2 RNAs found within symptomatic and asymptomatic individuals. Through the split-aligned reads that were not mediated from the TRS sites in the amplicon-seq data, we detected up to 10^4^ per million SARS-CoV-2 paired reads harboring TRS-independent junctions of at least 20 nucleotides in each sample. These deletion events were more prevalent in viral samples from symptomatic hosts (two-sided Wilcoxon rank-sum test, *p* = 2.3 × 10^−8^) (Fig. 3a), potentially due to more active viral replication in these hosts resulting in greater production of structural variants. In total, we detected 8551 unique deletions in viral RNAs that were supported by ≥2 independent reads. While the vast majority of them were sporadic events that occurred in isolated cases, 501 (6%) deletions were consistently observed in >10% of samples; either specific in symptomatic (*n* = 375), asymptomatic hosts (*n* = 38), or in both (*n* = 88) (Fig. 3b). It is interesting to note that, in symptomatic cases, these frequent structural deletions were not only more abundant but also significantly larger in sizes (median spans 198 vs. 46 nucleotides, *p* = 1.6 × 10^−15^), pointing to a potential selection force for different types of viral variants adapted in distinct cohorts of host responses. In all, 305 of 375 (81%) of the symptomatic vs. 8 out of 38 (21%) of the asymptomatic specific deletions were over 100 nucleotides, respectively. These deletion variants with large deletions could be potentially a subpopulation of defective interfering RNAs shown previously for other coronaviruses in the in vitro cultured cells during high MOI^27,34^.

These deletions were spread across the entire viral genome (Fig. 3c). To investigate the existence of distinct sets of deletions in viral RNAs selected in hosts with differences in disease severity, we examined their relative abundance (defined by normalized counts of read support) and frequencies (defined by the proportions of symptomatic vs. asymptomatic samples found). We revealed 296 deletions significantly enriched in the symptomatic and ten deletions in asymptomatic infections (*p* value <0.05) (Supplementary Data 5). Among them, 263 and nine deletions were exclusively found in symptomatic and asymptomatic specimens, respectively. We were particularly interested in the ten deletions preferentially found in the asymptomatic hosts (Fig. 3d) and their impact on the integrity of viral sgRNAs and proteins. Notably, three of them located within the coding regions of sgRNAs and two of the three deletions (42 and 82 nucleotides, respectively) affected the protein-coding region of sgRNA_ORF3a. These deletions were predicted to yield ORF3a protein variants with C-terminal extension and truncation (Fig. 3d). ORF3a protein was shown to induce apoptosis in infected cells^49^, an important host antiviral defense mechanism that controls the inflammatory response^50^. The alteration in the ORF3a protein could weaken its proapoptotic activities which potentially reduce apoptosis-mediated immune responses and result in milder or even asymptomatic infection. Moreover, additional deletion of 78 nucleotides in the open reading frame of nsp16, a cap-synthesizing methyltransferase required for viral replication^51,52^, was found in the asymptomatic patients carrying both ORF3a deletions. This deletion removes 26 amino acids in the NSP16 J-motif, removing β2 sheets and fusing α3 and α4 helices, responsible for ligand binding^53^. Other asymptomatic-associated deletions were found within the coding regions of sgRNA_N, nsp5 which were predicted to yield truncations in encoded proteins for a nucleocapsid and 3C-like proteinase, respectively. Taken together, the existence of different types of deletions in viral RNAs exclusively observed in multiple independent infected individuals exhibiting distinct host responses strongly implicate the functional significance of structural variants in conferring features of SARS-CoV-2 virulence and pathogenicity.

### Full-length Iso-seq analysis revealed extensive structural variation in SARS-CoV-2 genomes

**Fig. 4:**
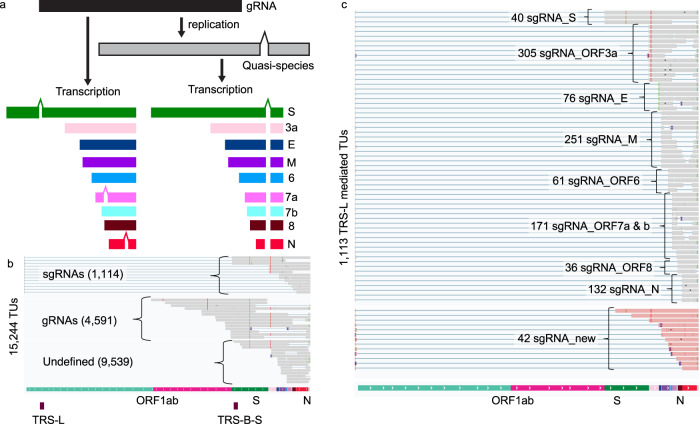
SARS-CoV-2 transcriptome diversity. **a** Proposed models of the origins of SARS-CoV-2 genomic deletions resulted from the lack of accurate replications of viral gRNAs (Right) or transcription of viral sgRNA (Left). **b** Assignment of full-length (FL) transcript units (TUs) revealed by long-read Iso-seq into viral sgRNAs (*n* = 1114), gRNAs (*n* = 4591), or undefined (*n* = 9539) based on their spans across transcription regulatory sequence (TRS) -Leader/-Body (TRS-L/TRS-B) junctions. **c** The distribution of FL TUs assigned to different sgRNA species based on their corresponding TRS-B sites.

**Fig. 5:**
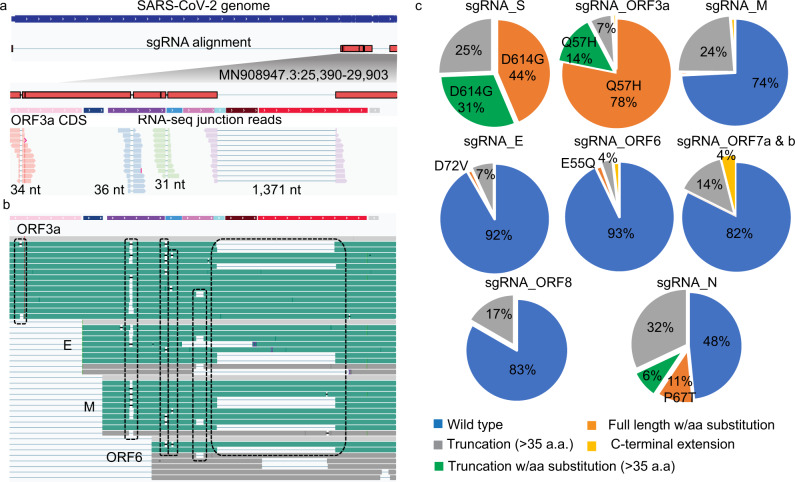
Phasing structural variants on subgenomic RNA (sgRNA) derived full-length (FL) transcript units (TUs) reveals SARS-CoV-2 proteome complexity. **a** An example of four deletions co-occurred on a sgRNA_ORF3a molecule uncovered by FL iso-seq analysis. **b** Examples show identical deletions (highlighted in boxes) detected in multiple FL cDNAs encoding different sgRNA species. **c** Distribution of predicted wild-type and mutant proteins encoded from the sgRNA-derived FL TUs.

The widespread and abundant deletions arisen in the symptomatic infections drew our attention to investigate their diversity and impact on viral sgRNA transcription. The observed viral variants presumably resulted from deletions occurring either during viral replication or transcription (Fig. 4a). To distinguish their origins and characterize their impact on the viral translated protein products, we examined these deletions in the context of their associated sgRNA structures by FL Iso-seq sequencing^54^. From ten samples with the highest ratio of SARS-CoV-2 content, we generated in total over two million high-quality FL cDNA sequences (Supplementary Data 6). Of which, 632,207 (31%) of them were SAR-CoV-2 origins and were further clustered into 15,244 distinct transcript units (TUs) supported by ≥2 FL cDNA sequences (Fig. 4b). Based on their alignments across TRS-L and their respective canonical TRS-B junction sites, 1114 FL TUs can be unambiguously assigned to sgRNA origins (Fig. 4c) while 4591 FL TUs aligned uninterrupted across TRS-B site and were determined as products from viral gRNAs (Fig. 4b). When we examined the presence of deletions in these FL TUs, the vast majority of the deletions were independently detected in both the sgRNA- and gRNA-derived FL TUs. The observed deletions were not resulted from the errors in sequences as no insertion or deletion was detected in the spike-in control reads produced from the same sequencing runs. Overall, from a total of 15,244 FL TUs, 3537 (23%) TUs harbored minimally one insertion or deletion over ≥20 bases, which raises the possibility that a substantial population of the SARS-CoV-2 virus carry structural variation during active infection. Their validity was further supported by the breakpoints inferred from the split reads in the metatranscriptome RNA-seq data. Of these 3573 TUs, 2873 (81%) TUs have at least one of their breakpoints supported by the split-aligned reads found in the metatranscriptome data and 2812 of these 2873 TUs (98%) have all of their breakpoints fully supported, suggesting that these were bona fide deletions occurred during viral gRNA replication as a result of low fidelity of RNA polymerases. These structural variants were subsequently propagated into protein-coding sgRNAs via transcription. Taking a TU of sgRNA_ORF3a as an example, this TU comprised four distinct deletions of 31, 34, 36, and 1371 nucleotides, respectively which were independently uncovered by short-read RNA-seq data (Fig. 5a). The same deletions can be also found in multiple TUs encoding distinct sgRNAs including sgRNA_E, _M, and _ORF6 (Fig. 5b). Therefore, structural variations of SARS-CoV-2 often lead to alternative sgRNA transcripts and substantial alterations in their translation products. These variants potentially exist as quasispecies to facilitate evolutionary selection and host adaptation as observed in other RNA viral species^55–58^.

### Structural variants in viral genomes further expand viral proteome complexity

Through placing the co-occurred insertions and deletions onto the individual FL transcripts, we can investigate the precise impacts of these variants on the viral protein translation. From the collection of the 1114 sgRNA-derived FL cDNA sequences, 23% of these transcripts carrying frameshifts with >35aa predicted translated protein products of truncations (20.1%), extension (1.2%), and new peptides of no known functional annotation (1.3%). Intriguingly, we also observed a low frequency of FL cDNAs producing potential fusion proteins. For example, a 257 amino acid membrane and ORF6 fusion peptide resulted from a 31-bases deletion. From the combinatorial effects of the non-synonymous SNVs and detected indels, the diversity of the SARS-CoV-2 encoded proteome were derived for each of the sgRNA species. We found the translated proteins as the following five groups: (1) Wild-type proteins of known annotation. (2) Proteins of known annotation with amino acid substitutions. (3) Truncated proteins of known annotation with or without amino acid substitutions. (4) Proteins of known annotation with C-terminal extension, and (5) New peptides. The validity of these protein variants was examined through comparison with the proteomic data generated by tandem mass spectrometry (MS) from SARS-CoV-2 infected cells^22^. Beyond the peptides translated from wild-type sgRNAs for S, 3a, M, 6, 7, 8, and N (Group 1), examples of variants (Fig. S3) from all groups except 4 can be found in the peptide MS spectra (Supplementary Data 7), further supporting the proteome complexity of the SARS-CoV-2 quasispecies. The proportions of the wild-type proteins and their corresponding variant types for each of the eight sgRNA-encoded proteins were shown in Fig. 5c. As expected, the vast majority of the predicted structural and accessory proteins translated from sgRNAs detected in these clinical samples were FL forms. Among the eight sgRNA-encoded proteins, ORF6 and Envelop are the most stable with 93 and 92% predicted FL wild-type proteins. It is noteworthy that 17% of the sgRNA_8 encoded truncated ORF8 protein products (Fig. 5c). Deletions in ORF8 were believed to reflect the adaptation to human host^59^ and were shown to confer an attenuation of replication during the epidemic of SARS-CoV-1^60,61^.

The predominant forms of S and ORF3a carry amino acid substitutions D614G and Q57H resulted from the non-synonymous SNVs in MN908947.3:25563 (G > U) and MN908947.3:23403 (A > G), respectively. SARS-CoV-2 D614G variant, emerging early during the pandemic, was suggested to possess higher infectivity^62^ while the effect of the Q57H variant on viral pathophysiology is currently less clear. Similar to D614G, the Q57H variant could be subjected to natural selection because it was only reported at <6% in Feb 2020^63^. 56% of Spike and 41% of Nucleocapsid were predicted to be truncated. We further annotated the deleted regions for functional domains using the NCBI CDD^41^ and, to our surprise, we found that 27, 41, and 42% of the predicted truncated Spike and Nucleocapsid proteins were lacking the N-terminal domain (NTD), receptor-binding domain (RBD), and RNA-binding domain (PSSM-ID 394862), respectively. S protein functions to mediate host cell entry through angiotensin-converting enzyme 2 (ACE2) receptor binding^64^ and the RNA-binding domain in N protein plays an important role in virus transcription and assembly^65^. These proteins are widely used as targets for vaccine and drug development^66^, with some exclusively targeting the NTD and RBD for treatment with neutralizing antibodies^67–69^. Deletions in the furin cleavage site (PRRA) and insertions in NTD of the S proteins were found to attenuate the pathogenesis and enable SARS-CoV-2 to escape the neutralizing antibodies^70–72^, highlighting the ability of SARS-CoV-2 to evolve and adapt host immune systems via structural plasticity. While the high frequencies of structural deletion in these proteins were only observed in selected samples with high viral content and most of these defective viral RNAs are unlikely to have the full capacity for proper translation or transcription, when putting under specific selection pressure, they could have significant ramifications on the efficacy of antibody-induced immunity and devising treatment strategies as shown in a recent study^72^.

## Discussion

In this study, we attempted to study the activity of SARS-CoV-2 transcription and the complexity of viral genome structural variation in infected human hosts with distinct disease severity. Through a combination of multi-scale genomic analyses, we quantitatively evaluated the expression of sgRNA species in a broad range of swabs collected for routine PCR-based diagnostics and revealed that the relative abundance of sgRNAs were significantly lower in the infected individuals without COVID-19 associated symptoms, indicating repressed viral transcription. The lower levels of sgRNAs detected in the asymptomatic infection was unlikely due to the timing of the sample collections, i.e, presymptomatic because sgRNAs are thought to be abundant in early infection^18^ and are stable as their presence can be detected for a significant time post active infection in symptomatic patients^21,73^. Moreover, the repression of sgRNA was not attributed to the differences in viral load because the sgRNAs quantities were normalized with the levels of gRNAs in each sample.

Different from diagnostic RT-qPCR assays which mainly measure the viral genomic RNA shedding, characterizing viral sgRNAs in the COVID-19 positive samples could be informative to understand the virus’ replicative activity in the host cells. Previous studies showed an increase of viral load is indicative of aggravation of symptoms^18^. The detection of sgRNAs in diagnostic samples was shown to correlate with active virus replication^18,74^ while others demonstrated the highly stable nature of sgRNAs, rendering them a poor marker of active infection^73^. Building from these observations, our results further show that sgRNA levels as assessed by the sgRNA/gRNA ratio are highly correlated with one measure of clinical severity, the presence of symptoms. The more rapid viral clearance seen in asymptomatic patients may result from successful host immune responses. Alternatively, it is plausible that the sgRNA production and the process to generate viral quasispecies are temporal in the asymptomatic infection. Future follow-up studies on the controlled time-course cases should reveal more insights on the kinetics of sgRNA transcription during asymptomatic infection. Our sgRNA findings suggest that RT-qPCR-based assays to quantitatively evaluate the relative abundance of sgRNAs may be a predictive measure of the clinical severity of COVID-19 symptoms. These results could have a significant impact on the conservation of medical resources during the rapid community spreading, much like what we are experiencing globally in recent weeks.

The structural plasticity we observed is attributed by the typical characteristics of RNA-virus quasispecies^75,76^. The diversity and biological roles of quasispecies have been actively studied in RNA viruses^28,29^. Mostly reported in the context of SNVs, these viral quasispecies, rather than merely background noise, could function as a population to have significant ramification in antigenic variability, viral fitness, and pathogenicity as many reports have shown^30–32,77–79^. Unfortunately, most of the genomic sequencing studies done on the SARS-CoV-2 clinical samples have focused on the consensus sequences^80^ and the structural diversity has been largely overlooked. Our study, extending from these findings, characterized the inter- and intra-patient variants in the form of structural deletions in the clinical specimens collected for routine diagnostic purposes. We showed distinct and recurring sets of viral RNA deletions in both symptomatic and asymptomatic infections and characterized the diverse structural variants associated with disease severity. Their consistent and preferential detection in multiple COVID-19 positive cases points to genome instability as a source of viral proteome complexity and potential evolutionary selection for host adaptation. While these observations could have potential clinical implications in the control and treatments of SARS-CoV-2, the functional significance of these quasispecies in the adaptation with the host immune system in the course of infection remains to be elucidated and should be a follow-up study. Taken together, when associated together with the host genetics and immune response, the sgRNA expression and structural diversity could provide insight in understanding host-viral interactions, evolution, and transmission. This, in turn, will guide risk mitigation, testing strategies, and inform future vaccine development.

### Reporting Summary

Further information on research design is available in the Nature Research Reporting Summary linked to this article.

## Supplementary information

Supplementary Data 1

Supplementary Data 2

Supplementary Data 3

Supplementary Data 4

Supplementary Data 5

Supplementary Data 6

Supplementary Data 7

Supplementary Data 8

Supplementary Information

Reporting Summary

Description of Additional Supplementary Files

## Data Availability

All data described in this study have been deposited in National Center for Biotechnology Information Sequence Read Archive under accession PRJNA690577. Source data for the main figures in the manuscript is available as Supplementary Data 8.
