# Supplementary material for: Reduced subgenomic RNA expression is a molecular indicator of asymptomatic SARS-CoV-2 infection
Source: Commun Med (Lond). 2021 Sep 22;1:33. doi: 10.1038/s43856-021-00034-y (PMC9053197; doi:10.1038/s43856-021-00034-y)
Supplement: Supplementary file 9 — Supplementary Information [file 43856_2021_34_MOESM9_ESM.pdf]

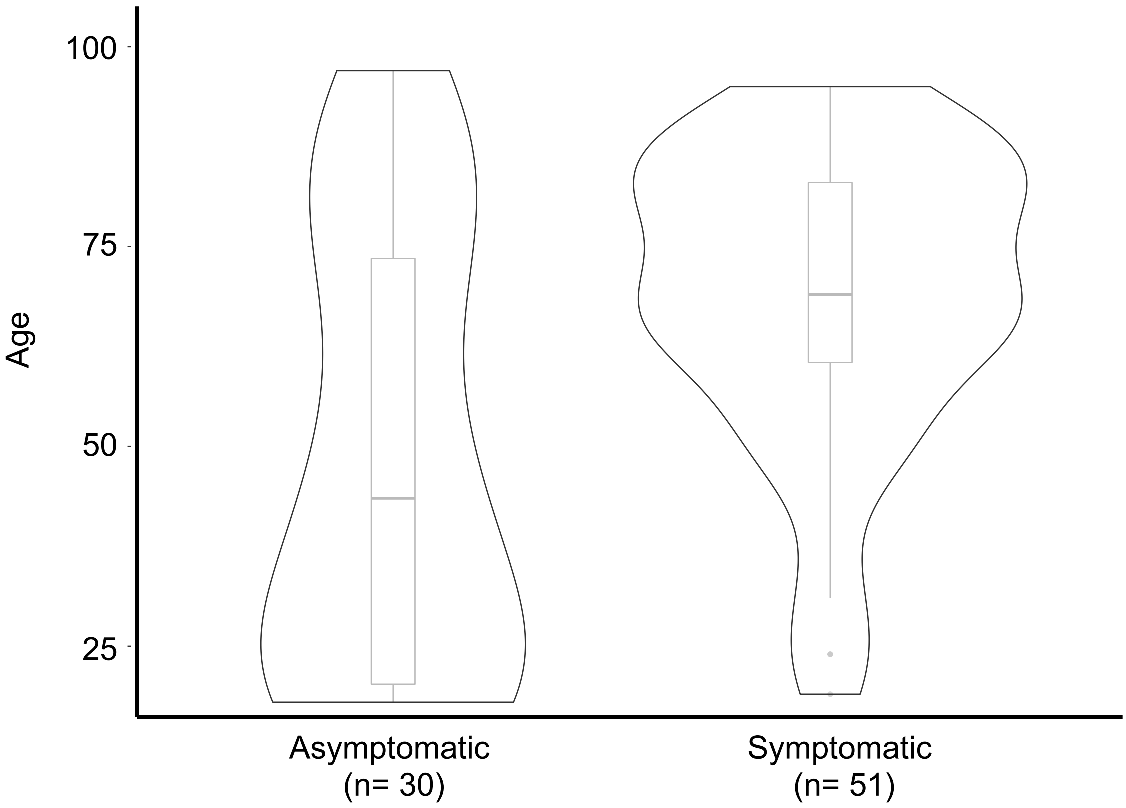

Figure S1: Age distributions of asymptomatic and symptomatic COVID-19 positive patients. Center line, median; boxes, first and third quartiles; whiskers, 1.5 × the interquartile range; points, outliers.

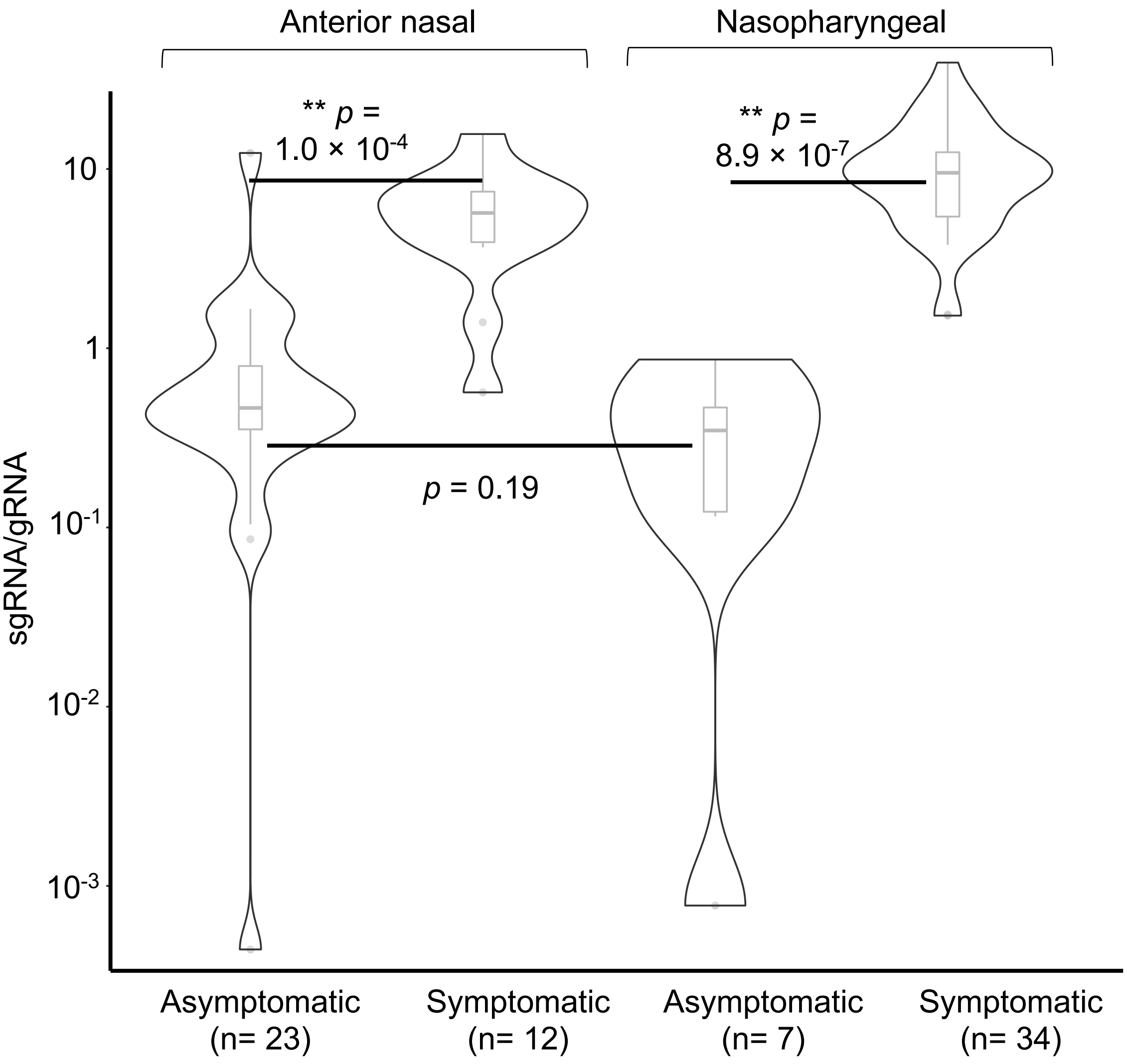

Figure S2: The normalized sgRNA abundance (sgRNA/gRNA) distribution of symptomatic and asymptomatic patients amongst the specimen types. Pairwise two-sided Wilcoxon Rank Sum Test with Bonferroni correction. Center line, median; boxes, first and third quartiles; whiskers, 1.5 × the interquartile range; points, outliers.

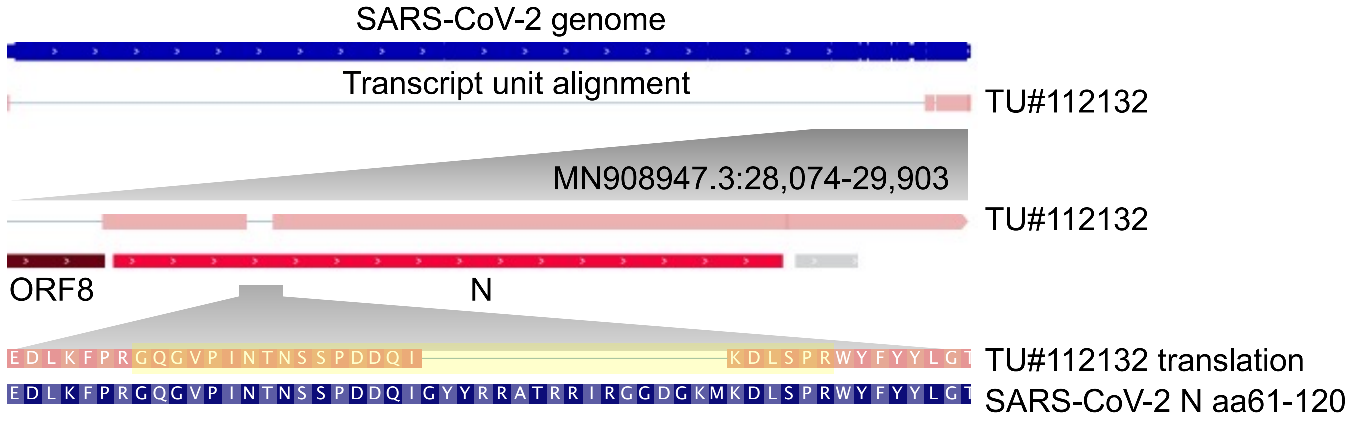

Figure S3: Nucleocapsid protein variant identified in tandem mass spectrometry data. Identified unique peptide GQGVPINTNSSPDDQIKDLSPR (indicated by yellow highlight) in tandem mass spectrometry data from translation of full-length Nucleocapsid transcript unit TU#112132, which identified deletion aa 85-101 with respect to canonical Nucleocapsid protein.
