# Supplementary material for: Reduced subgenomic RNA expression is a molecular indicator of asymptomatic SARS-CoV-2 infection
Source: Commun Med (Lond). 2021 Sep 22;1:33. doi: 10.1038/s43856-021-00034-y (PMC9053197; doi:10.1038/s43856-021-00034-y)
Supplement: Supplementary file 11 — Description of Additional Supplementary Files [file 43856_2021_34_MOESM11_ESM.pdf]

Description of Additional Supplementary Files

File Name: Supplementary Data 1

Description: Sample summary

File Name: Supplementary Data 2

Description: Amplicon-sequencing primer sequences

File Name: Supplementary Data 3

Description: Amplicon-Seq data summary

File Name: Supplementary Data 4

Description: Poly-A+ RNA-Seq data summary

File Name: Supplementary Data 5

Description: Deletions enriched in the asymptomatic and symptomatic samples

File Name: Supplementary Data 6

Description: Iso-Seq data summary

File Name: Supplementary Data 7

Description: Identified peptides unique to Iso-Seq full-length transcript units (TUs) translation

File Name: Supplementary Data 8

Description: Source data
